# Uncovering the relationship between gut microbial dysbiosis, metabolomics, and dietary intake in type 2 diabetes mellitus and in healthy volunteers: a multi-omics analysis

**DOI:** 10.1038/s41598-023-45066-7

**Published:** 2023-10-20

**Authors:** Mohammad Tahseen Al Bataineh, Axel Künstner, Nihar Ranjan Dash, Habiba S. Alsafar, Mohab Ragab, Franziska Schmelter, Christian Sina, Hauke Busch, Saleh Mohamed Ibrahim

**Affiliations:** 1 Department of Genetics and Molecular Biology, College of Medicine and Health Sciences, Khalifa University of Science and Technology, PO Box 127788, Abu Dhabi, UAE; 2 Center for Biotechnology, Khalifa University of Science and Technology, Abu Dhabi, UAE; 3 Lübeck Institute of Experimental Dermatology, University of Lübeck, 23562 Lübeck, Germany; 4 Institute for Cardiogenetics, University of Lübeck, 23562 Lübeck, Germany; 5 Department of Clinical Sciences, College of Medicine, University of Sharjah, Sharjah, UAE; 6 Institute of Nutritional Medicine, University of Lübeck, Lübeck, Germany

**Keywords:** Microbiome, Clinical microbiology

## Abstract

Type 2 Diabetes Mellitus has reached epidemic levels globally, and several studies have confirmed a link between gut microbial dysbiosis and aberrant glucose homeostasis among people with diabetes. While the assumption is that abnormal metabolomic signatures would often accompany microbial dysbiosis, the connection remains largely unknown. In this study, we investigated how diet changed the gut bacteriome, mycobiome and metabolome in people with and without type 2 Diabetes.1 Differential abundance testing determined that the metabolites Propionate, U8, and 2-Hydroxybutyrate were significantly lower, and 3-Hydroxyphenyl acetate was higher in the high fiber diet compared to low fiber diet in the healthy control group. Next, using multi-omics factor analysis (MOFA2), we attempted to uncover sources of variability that drive each of the different groups (bacterial, fungal, and metabolite) on all samples combined (control and DM II). Performing variance decomposition, ten latent factors were identified, and then each latent factor was tested for significant correlations with age, BMI, diet, and gender. Latent Factor1 was the most significantly correlated. Remarkably, the model revealed that the mycobiome explained most of the variance in the DM II group (12.5%) whereas bacteria explained most of the variance in the control group (64.2% vs. 10.4% in the DM II group). The latent Factor1 was significantly correlated with dietary intake (*q* < 0.01). Further analyses of the impact of bacterial and fungal genera on Factor1 determined that the nine bacterial genera (*Phocaeicola, Ligilactobacillus, Mesosutterella, Acidaminococcus, Dorea A, CAG-317, Caecibacter, Prevotella* and *Gemmiger*) and one fungal genus (*Malassezia furfur*) were found to have high factor weights (absolute weight > 0.6). Alternatively, a linear regression model was fitted per disease group for each genus to visualize the relationship between the factor values and feature abundances, showing Xylose with positive weights and Propionate, U8, and 2-Hydroxybutyrate with negative weights. This data provides new information on the microbially derived changes that influence metabolic phenotypes in response to different diets and disease conditions in humans.

## Introduction

Dietary fiber intake is crucial for maintaining general health. Several research groups have linked a high-fiber diet with reduced risks of many health conditions^1,2^. Numerous health advantages, such as better blood glucose control, cardiovascular health, weight management, and digestive function, have been linked to consuming a high-fiber diet^3^. Dietary fibers' impacts on health outcomes are mediated in a significant way by the gut microbiome, which contains bacteria like *Prevotella*. *Prevotella*, which is well-known for its capacity to convert dietary fiber into advantageous short-chain fatty acids, produces SCFA which have been shown to be advantageous in maintaining gut homeostasis^4,5^. However more investigation is required to properly understand the complex connection between fiber consumption, the gut microbiome, and particular bacterial taxa like *Prevotella* in the context of health and diabetes treatment. Type 2 Diabetes Mellitus (DM II) is a rising health concern worldwide, involving almost 6.3% of the world’s population and causing more than 1 million deaths annually^6^. The Middle East and North Africa region have the second-highest rate of worldwide diabetes growth, with a projected 96.2% increase in diabetes cases by 2035^7^. In addition, lifestyle, obesity, nutrition, and environment, among other factors, gut microbiota dysbiosis has become recognized as a significant diabetes-related cause. However, the exact link between DM II and gut microbiota composition is yet unclear. Currently, most studies have gathered evidence for the role of gut bacteriome in the onset and progression of DM II, though reports vary regarding the association of particular taxonomic groups with the disease^8^. Interestingly, a recent study reported that different microbes were associated with the same metabolic outcomes in other geographical areas^9^. While much emphasis is focused on the role of gut bacteria in DM II, the effect of gut mycobiome (fungal species) and metabolome in DM II are poorly explored and understood.

When examining the potential molecular mechanisms by which the gut microbiota changes during the disease process in type 2 diabetes (DM II), studies have shown that gut inflammation, compromised gut permeability, impaired glucose and lipid metabolism, insulin insensitivity, increasing fatty acid oxidation, and interactions with dietary components were the main potential modulating factors^8,10,11^.

Our understanding of the complex relationship between gut microbiota and DM II has been chiefly due to the technological advancements in omics studies such as metagenomics, metabolomics, proteomics, and transcriptomics. Despite the variation in bacterial species, there is a functional congruity that has helped to dissect the roles and actions of genes, proteins, and molecules in cellular metabolism in the context of metabolomic profiling. Metabolomics is especially valuable to trace the compounds produced by the bacteria in response to the host, particularly fungal species due to the increasing evidence of their interactions with the microbiome and the host at large. Now we can integrate and apply multi-omics approaches to explore the connection between microbiota dysbiosis and abnormal metabolic signatures in DM II to improve our understanding of the disease process, identification of biomarkers and efficient therapeutics, and application of precision medicine^12^. The specific aim of this study is to explore the impact of diet on the composition of the gut bacteriome, mycobiome, and metabolome in individuals with and without type 2 diabetes, with a particular focus on understanding the connections between microbial dysbiosis and abnormal glucose homeostasis. we evaluated 16S rRNA and ITS2 sequence data and metabolic profiles of individuals from the United Arab Emirates (UAE) with or without DM II using stool samples and food questionnaires to determine dietary fiber intake.

## Material and methods

### Patient inclusion and ethical statement

Following the acquisition of ethical approval from the University Hospital Sharjah Ethics Research Committee (UHS-HERC-021-0702), the study was conducted in adherence to the relevant research guidelines and regulations specified by the committee. We randomly identified 25 subjects diagnosed with DM II at the endocrinology clinic. Additionally, 25 otherwise healthy Emirati individuals (HbA1C level < 6%) were recruited as healthy controls. All subjects were given an information sheet and signed a written informed consent form. The demographic information such as age, gender, and diet (using a validated dietary fiber intake short food frequency questionnaire (DFI-FFQ)) was collected. The high dietary fiber intake cut-offs were > 25 g/day for females and > 30 g/day for males, and low dietary fiber intake cut-offs were below 17.5 g/day for females and 22.1 g/day for males^13^. Demographic information is given in Supplementary Table 1.

### Bacterial and fungal sequencing

Bacterial and fungal DNA extraction and sequencing were already described in Al Bataineh et al*.*^19^. Briefly, DNA extraction of fecal samples was done using QIAamp PowerFecal DNA Kit (Qiagen Ltd., GmbH, Germany) following the manufacturer’s instructions (Qiagen Ltd.). Bacterial 16S rRNA gene amplification was performed targeting the V4 region with dual barcodes and sequenced with an Illumina MiSeq using 250bp paired-end kit (v.2)^14^. ITS2 region was sequenced on an Illumina MiSeq using a dual barcoding protocol (250bp paired-end sequencing)^14^.

### Sample preparation for metabolic profiling

In brief, 100 mg of fecal samples were homogenized in 500 µl of water Optigrade for HPLC (Promochem SO-6795-B025). Then, samples were incubated for 15 min at 20 °C, 1000 rpmThe mixture was centrifuged for 15 min at 14 000 rpm, 4 °C. The supernatants (200 µl) are evaporated to dryness using a SpeedVac. For NMR analysis, the dried extracts are subsequently reconstituted in 200 µL phosphate buffer in D_2_O (100 mM, pH 7.4) containing 0.1 mM TSP-d4. The whole sample volume is transferred into a 3.0 mm NMR tube used for the NMR analysis.

### NMR spectroscopy

For each sample, one-dimensional ^1^H-NMR spectra were acquired on a Bruker 600 MHz Avance III HD spectrometer (Bruker BioSpin with TopSpin 3.5pl7) operating at 600.13 MHz proton Larmor frequency and equipped with a 5 mm PA TXI 1H-13C-15N and 2H-decoupling probe including a z-axis gradient coil, an automatic tuning-matching and an automatic sample changer (SampleJet). The temperature was kept stable within 0.1 K using a BCU I. Before starting measurements manually, samples were kept inside the NMR probe head for at least 5 min to equilibrate temperature at 300 K. The standard Nuclear Overhauser Effect SpectroscopY (NOESY) presat pulse sequence (noesygppr1d; Bruker BioSpin) was used to detect both signals of small metabolites and high molecular weight macromolecules. The parameters of the experiment were: 512 scans, 32,768 data points, a spectral width of 12.0166 ppm, an acquisition time of 2.27 s, a relaxation delay of 2 s, and a mixing time of 0.01 s. Fourier-transformed spectra were automatically corrected for phase and baseline distortions using Topspin 3.2 (Bruker BioSpin) and then automatically calibrated to the proton signal of TSP-d4 at 0.00 ppm.

### Metabolic profiling

Metabolite identification was made using the SBASE database in Amix (v3.9.11; Bruker BioSpin, Germany) or available assignments in the literature. The peaks of the identified metabolites were fitted by a combination of a local baseline and Voigt functions based on the multiplicity of the NMR signal. The absolute concentration of each metabolite was calculated according to the equation described by Serkova et al.^15^. Metabolites below the limit of detection were set to zero.

### Data processing, data import and filtering

Raw sequencing data from the bacterial assay (*fastq* format) was demultiplexed and processed into amplicon sequence variants (ASVs) using dada2 (v1.24.0)^16^. In brief, the expected error rate was set to 1 for the forward and to 2 for the reverse read. The minimum read length after trimming low-quality bases was set to 200bp; shorted read pairs were discarded. Next, forward and backward reads were merged into contigs and contigs were size selected (between 252 and 253 bp). Afterward, chimeric sequences were removed following the dada2 recommendations and IdTaxa (DECIPHER package (v2.18.1)) with GTDB r207 as the reference database was used for taxonomic assignments^17,18^. ASVs not belonging to the kingdom Bacteria or with unassigned phylum were excluded from further analysis. Additionally, ASVs belonging to the phylum *Cyanobacteria* were removed if there were annotated as unknown at class level.

Bacterial ASVs from dada2 (described above) and fungal data (processed data, OTU table and taxonomic assignments, was retrieved from Al Bataineh et al*.*^19^), were imported together with the metabolite assay into R (v4.2.2, phyloseq v1.40.0)^19,20^. The fungal assay was screened for operational taxonomic units (OTUs) with unknown or unassigned phyla, which were excluded from further analysis. Additionally, OTUs belonging to the order *Talaromyces* were removed as potential contaminants because they are not part of the normal gut flora. Samples with missing assay data were removed, resulting in 41 samples with complete data (20 control samples, 21 Type 2 Diabetes Mellitus samples).

### Alpha and beta diversity

Alpha diversity was estimated on ASV/OTU/metabolite level for each sample and assay using Shannon’s index, and significance was assessed using non-parametric Wilcoxon tests. To estimate beta diversity, abundances (counts) were *centered log-ratio* transformed (*clr*), and distances were calculated using Euclidean distance (i.e., Aitchison distance). The *clr* values are scale-invariant and sequencing depth does not play a role in the downstream analysis using these values. Permutational multivariate analysis of variance using distance matrices (PERMANOVA) was used to compare differences in beta diversity (*adonis2* function, as implemented in the vegan package v2.6-4, with 99,999 permutations). In detail, the following models were used:

$$\begin{aligned} & beta_{Bacteriome} \sim Diet + Disease + Prevotella + Age + Gender + BMI + Diet:Disease \\ & beta_{Mycobiome} \sim Diet + Disease + Prevotella + Age + Gender + BMI + Diet:Disease \\ & beta_{Metaobolome} \sim Diet + Disease + Prevotella + Age + Gender + BMI + Diet:Disease \\ \end{aligned}$$

Diet (low or high fiber intake), disease (healthy or Type 2 Diabetes Mellitus) and gender (male or female) are categorial variables and the remaing factors are continuous variables.

### Differential abundance testing

Differential abundant (DA) taxa (genera) and metabolites were identified using multiple DA methods to ensure robust biological findings. Note, differential abundance testing was performed within the healthy control group and within the DM II group separately and not between the healthy and the DM II group. For all three assays, we used ALDEx2 (v1.28.1), ANCOM-BC (v1.6.0), and MaAsLin2 (v1.10.0). Additionally, dacomp (v1.26) was applied to assays where count data was available (bacterial and fungal assays)^21–25^. Genera and metabolites with low prevalence (< 20%) were removed from the data before DA testing. ALDEx2 was run with 512 Monte Carlo instances to estimate the underlying distributions, and Welch’s t-test statistic. ANCOM-BC was run with standard parameters except for the detection of structural zeros (set to TRUE), the maximum number of iterations for the E-M algorithm (set to 10,000), and we used a conservative variance estimate of the test statistic as it is recommended for small sample sizes (conserve option set to TRUE). Settings for MaAsLin2 were according to Nearing et al.^29^ using total sum scaling (TSS) to normalize the data followed by arcsine square rooted transformation^26^. The resulting p-values from ALDEx2, ANCOM-BC and MaAsLin2 were corrected for multiple testing using Benjamini–Hochberg correction. The dacomp method was run with standard parameters using a Wilcoxon rank-sum test and a discrete false-discovery rate to correct for multiple testing. Results from DA testing were weighted using the number of algorithms that detected a genus/metabolite as differential abundant (*fdr* < 0.1), and a genus/metabolite with a weight of 2 or higher was considered significant.

### Correlation analysis and (semi-) unsupervised learning

To calculate correlations and for (semi-) unsupervised learning data *clr* transformed counts of highly variable features (genera or metabolites with a variance larger than 10) were used.

Pairwise correlations between all three assays were calculated using the *cor.mtest* function (corrplot v0.92) and results were filtered for significant correlations (*p* < 0.01, absolute Spearman’s ρ > 0.3).

To uncover sources of variability within each group (healthy or disease) we performed a multi-omics factor analysis as implemented in MOFA2 (v1.6.0)^27^. The data was imported and samples were labeled (Control or DM II) to be able to compare the sources of variability within each group. Next, the method was run with 10,000 iterations in ‘slow’ convergence mode to ensure model convergence and seed set to 42; the final model converged after 8466 iterations. Significantly associated features were fitted using the following linear model:

$$Abundance_{CLR} \sim factor\_values\_of\_group\;({\text{with group either healthy control or DMII}})$$

To look into differences between disease states a semi-supervised learning approach that performs feature extraction from noisy and high-dimensional data as implemented in KODAMA (v2.4) was applied to stratify the data by disease and diet with partial least squares discriminant analysis (PLS-DA) as classifier function. The best number of classifiers was determined using entropy filtering for models with 2, 4, 6, 8, 10, 20, 50, and 100 parameters. The model with the lowest entropy was selected for further analysis, and linear regression was applied to the best model results to estimate the effect of diet on estimates of the first and second dimensions.

### Statistical analysis and visualization

All analyses were performed using R (v4.2.2). For data handling the phyloseq library and tidyverse (v1.3.2) were used; cowplot (v1.1.1), ggpubr (0.5.0), and patchwork (v1.1.2) were applied to create the figures. Gradient boosting was performed for each group (control or DM II) separately using the packages caret (v6.0-93) and gbm (v2.1.8.1) with a fivefold cross-validation approach. Briefly, the data were re-coded to match the Bernoulli distribution (low/high dietary fiber intake). Next, the data was repeatedly split into training (80%) and test data (20%) (1000 iterations), and for each iteration, the maximum number of trees explored was set to 20,000, the *class.stratify.cv* argument was set to TRUE, and the shrinkage parameter applied to each tree in the expansion was set to 0.001. The code used for the microbiota analysis is available at https://github.com/kunstner/2022_Fiber_DMII_Middle_East.

## Results

After processing the data, 20 control samples and 21 Type 2 Diabetes Mellitus samples contained data from all three collected assays (bacteriome, mycobiome, metabolome). The bacteriome data comprised on average 9534 contigs (s.d. ± 3465), ranging from 2032 to 18,320 contigs (786 ASVs). The mycobiome data contained on average 5450 contigs (s.d. ± 4964), with a minimum of 532 and a maximum of 20,605 contigs (460 OTUs).

### Data stratification

Age was significantly different between control and Type 2 Diabetes Mellitus (DM II) samples (control 27.1 ± 7.5, DM II 60.4 ± 10.8; Wilcoxon test *p* = 8.70*10^–8^; see Suppl. Figure 1A for details). Additionally, we found a significant difference in BMI between the two groups (*p* = 6.04*10^–4^) with higher BMI in the DM II group (control 24.4 ± 5.05; DM II 30.8 ± 6.76). Due to the significant differences in age and BMI between controls and DM II samples, data were stratified by disease status to avoid findings linked to differences in age and BMI and to ensure that findings are related to fiber intake. After splitting the samples into control and DM II samples (control: low fiber diet n = 11, high fiber diet n = 9; DMII: low fiber n = 9, high fiber diet n = 12) no significant differences in age were observed within each data set between low and high dietary fiber intake (healthy: *p* = 0.52; DMII *p* = 0.27; Suppl. Figure 1B), and BMI was similar within each subset with respect to fiber intake (*p*_*control*_ = 0.09, *p*_*DMII*_ = 1.00).

### Diversity analysis

To evaluate differences in diversity we estimated sample-wise diversity (alpha diversity) and the variation of microbial communities between samples (beta diversity).

**Figure 1:**
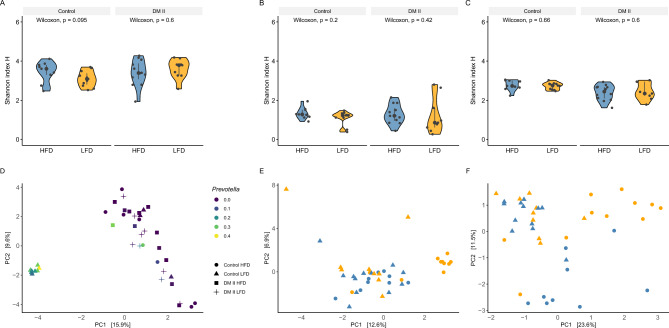
Alpha diversity estimated using Shannon’s index for bacteriome (**A**), mycobiome (**B**), and metabolite data (**C**). Distance-based analysis (beta diversity) of diversity (Aitchison distance) for bacteriome with *Prevotella* abundances color-coded is shown in (**D**). (**E, F**) Beta diversity for the mycobiome, and metabolite data, respectively; controls (n = 20) are encoded using dots, and triangles encode DM II individuals (n = 21); high dietary fiber intake (n_Controls_ = 9, n_DMII_ = 12) is colored blue, and low dietary fiber intake (n_Controls_ = 11, n_DMII_ = 9) is colored by orange.

Alpha diversity, estimated by Shannon’s index (*H*), showed no significant differences in any of the assays when comparing a high dietary fiber diet (HFD) versus a low dietary fiber diet (LFD) in the healthy control group or in the DM II group (Wilcoxon test, *p* > 0.05; Fig. 1A–C).

Next, we looked at the abundances of the genus *Prevotella* because of its known association with fiber intake (Suppl. Figure 2 shows Prevotella abundances for our data)^28^ and we link the abundances to alpha diversity. We found a significant increase in the LFD group compared to the HFD group in the control samples but not in DM II samples (likelihood ratio test: *H*_*0*_ = *1*; *H*_*1*_ = *diet*; *p*_*control*_ = 0.0051, *p*_*DMII*_ = 0.9856). Furthermore, no general correlation was observed between Shannon’s *H* and *Prevotella* abundances (Spearman’s ρ = − 0.2449, *p* = 0.1228).

To investigate factors that have potentially an impact on the community structure (beta diversity), the data were not stratified by disease, to be investagte differences between diseased and healthy samples as well. Disease (PERMANOVA; bacteria: *R*^2^ = 0.0524, *p* = 8.3 × 10^–4^; fungal: *R*^2^ = 0.0536, *p* = 1.2 × 10^–4^; metabolite: *R*^2^ = 0.1307, *p* = 1.0 × 10^–5^) and diet (bacteria: *R*^2^ = 0.0487, *p* = 2.2 × 10^–3^; fungal: *R*^2^ = 0.0443, *p* = 1.9 × 10^–3^; metabolite: *R*^2^ = 0.0568, *p* = 3.6 × 10^–3^) contribute significantly to differences in community structure in all three assays. Additionally, in the bacterial assay, we found significant associations with *Prevotella* abundances (*R*^2^ = 0.0654, *p* = 3.0 × 10^–5^). *Prevotella* abundances were significantly correlated with the first and the second principal component (ρ_PC1_ = − 0.3514, *p*_PC1_ = 0.0243; ρ_PC2_ = − 0.4966, *p*_PC2_ = 9.6 × 10^–4^) showing its influence on community structure in the data. In the fungal assay, *Prevotella* abundances (*R*^2^ = 0.0371, *p* = 1.3 × 10^–2^) had an additional significant effect on beta diversity besides the already mentioned factors disease and diet. Beta diversity for each assay is shown in Fig. 1D–F and PERMANOVA results are shown in Supplementary Tables 2–4.

### Taxonomic and metabolite abundances

**Figure 2:**
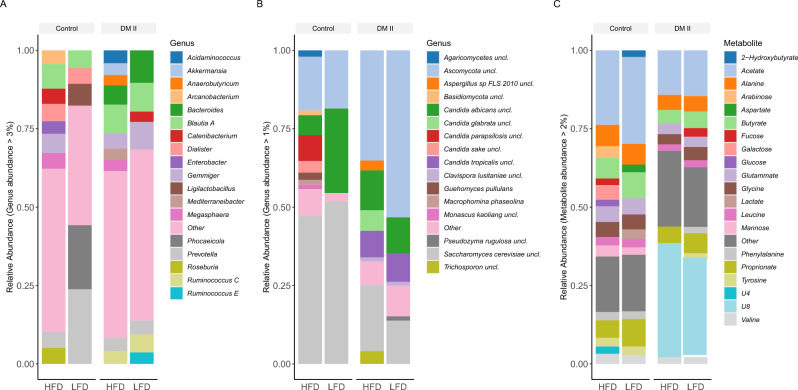
Relative abundances of (**A**) bacterial genera (abundances above 2%), (**B**) fungal genera (above 1%), and (**C**) metabolites (above 2%) for healthy controls and DM II samples.

The top five most abundant bacterial genera were *Prevotella*, *Phocaeicola*, *Blautia A*, *Dialister*, and *Bacteroides*. In the fungal data, we found *Ascomycota uncl.*, *Saccharomyces cerevisiae uncl.*, *Candida albicans uncl.*, *Candida tropicalis uncl.*, and *Clavispora lusitaniae uncl.* as the top 5 genera. The unknown metabolite 8 (U8), Acetate, Propionate, Butyrate, and Alanine showed the highest abundances in the metabolite data. Genus and metabolite abundances for each group are shown in Fig. 2, and phylum abundances are shown in Suppl. Figure 3.

The bacterial genera *Coprococcus A, Dorea A, Dysosmobacter, Gemmiger, Lachnospira* and *Sutterella* showed a significant increase in the HFD samples, whereas *Acidaminococcus, Bifidobacterium, CAG-317, Lachnoclostridium B, Ligilactobacillus, Mesosutterella, Mitsuokella, Phocaeicola and Prevotella* were higher abundant in the LFD samples when considering the control group (*fdr* < 0.1; Suppl. Figure 4). In the fungal assay, no genus was significantly different between HFD compared to LFD in controls (*fdr* > 0.1). The metabolites Propionate, U8, and 2-Hydroxybutyrate were significantly lower in HFD and 3-Hydroxyphenyl acetate was significantly higher in HFD compared to LFD in controls (*fdr* < 0.1; Suppl. Figure 5). In the DM II group, no genera (bacterial or fungal) or metabolites were identified as differentially abundant between HFD and LFD. All significantly different genera/metabolites are shown in Supplementary Table 5.

### Correlation between bacterial, fungal, and metabolite assay

To uncover two-dimensional relationships, Spearman rank correlations of *centered log-ratio* transformed (*clr*) abundances between the three assays (bacteria, fungal, metabolite) were calculated for features with a variance above 10. As already pointed out, *clr* transformed abundances are scale-invariant and therefore independent from sequencing depth. Correlations were considered significant if *p*-values were below a threshold of 0.01 and an absolute value of Spearman’s ρ above 0.3. The two diets showed markedly different patterns of correlations, regardless of the disease group (Suppl. Figure 6 and 7). Particularly, the correlations between bacterial and fungal data were dominated by negative correlations between *Meridosma* and several fungal genera in control LFD samples (e.g., *Candida dubliniensis uncl.*, *Malassezia restricta*). The metabolite Glycine showed a strong negative correlation with several bacterial genera in the control HFD group (e.g., *Clostridium AP/AQ/P*, *Lactobacillus*, *Streptococcus*), and the metabolite galactose showed negative associations with some fungal genera in the control HFD group. In contrast, DMII HFD correlations were dominated by positive associations of *Odoribacter* with several fungal genera (e.g. *Basidiomycota uncl.*, *Pichia manshurica uncl.*). In addition, the LFD DMII group showed marked negative correlations between Propionate and several bacterial genera (e.g. *Pseudoruminococcus A*, *Victivallis*).

### Multi-omics factor analysis

**Figure 3:**
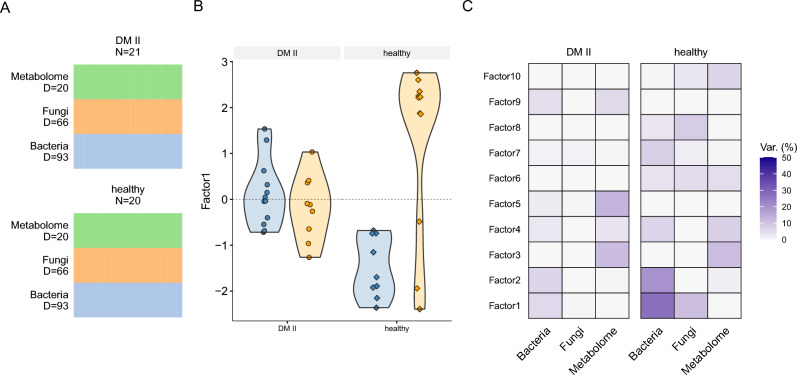
Graphical summary of the number of samples (‘N’) and features per assay (‘D’) used for the multi-omics factor analysis per group; the number of features is the same between groups (**A**). Values for latent Factor1 stratified by the disease are shown in (**B**), where blue refers to high and orange to low dietary fiber intake, respectively. (**C**) The proportion of variance explained by each assay for the DM II and healthy controls.

Next, we tried to uncover sources of variability that drive each of the groups using multi-omics factor analysis (MOFA2) with ten factors on the full data consisting of the three different assays (bacterial, fungal, and metabolite) and all samples combined (control and DM II). The data were filtered for highly variable features per assay as we did in the correlation analysis to ensure the same number of features per assay and group (Fig. 3A). From the high dimensional input data, MOFA2 infers a set of latent factors in an interpretable low-dimensional representation. These latent factors represent the main sources of variation across data sets and can be used to discriminate subgroups within the data.

The bacteriome explained most of the variance in the control group (64.2% *vs* 10.4% in the DM II group), whereas the mycobiome explained most of the variance in the DM II group (12.5%), which was very similar in the control group (12.3%). The metabolome contribution was higher in the control group (13.7%) compared to the DM II group (5.8%). Ten latent factors were identified, capturing global sources of variability in the data. To perform variance decomposition, each latent factor was tested for significant correlations with age, BMI, diet, and gender. Latent Factor1 was significantly correlated with dietary fiber intake (*q* < 0.01) in the combined data. Stratifying the data by disease revealed that latent Factor1 is significantly correlated with fiber intake in the control group as well (*-log*_*10*_*(q)* = 3.62). Additionally, gender was associated with latent Factor10 in the control group (*-log*_*10*_*(q)* = 2.02). The DM II group showed no significant association with fiber intake, gender, age, or BMI (*q* > 0.01). All significant associations are shown in Supplementary Fig. 8, and estimated values for latent Factor1 are shown for each sample stratified by disease and dietary fiber intake in Fig. 3B.

The major source of variation was captured in latent Factor1 and this factor was found to be linked to diet in the full data and in the control group. Therefore, the contribution of the bacteriome, mycobiome, and metabolites on this factor was further investigated, stratified by disease status. In the control group, most of the variance was explained by the bacteriome (29.3%), followed by the metabolite (4.3%) and the mycobiome data (3.8%) (Fig. 3C). In the DM II group, bacteria explained 1.4% and fungal and metabolite less than 1% each. In the following, we further analyze the impact of bacteria and fungi genera on latent Factor1. Nine bacterial genera (*Phocaeicola, Ligilactobacillus, Mesosutterella, Acidaminococcus, Dorea A, CAG-317, Caecibacter, Prevotella* and *Gemmiger*; Suppl. Figure 8) and one fungal genus (*Malassezia furfur*; Suppl. Figure 9) were found to have an estimated absolute weight of 0.6 or higher. Four metabolites showed an absolute weight above 0.6 (U8, 2-Hydroxybutyrate and Propionate with negative weights; Xylose with positive weights; Suppl. Figure 10).

A linear regression model was fitted per disease group for each genus to visualize the relationship between the factor values and feature abundances. Absolute R-values of the models were significantly higher in the control group than in the disease group (one-sided Wilcoxon test, *p* = 2.9 × 10^–5^). In the bacteriome, most of the associations are similar with respect to the sign of the R-value between control and DMII samples (7 out of 9 comparisons). In the mycobiome data (genus *Malassezia furfur*), the healthy control group showed negative and the DM II group showed positive association with estimated values for Factor1 (*R*_*control*_ = − 0.69, *R*_*DMII*_ = 0.18). The four metabolites showed a higher correlation with Factor1 in the control group compared to the DMII group.

### Confirmation of multi-omics factor analysis

**Figure 4:**
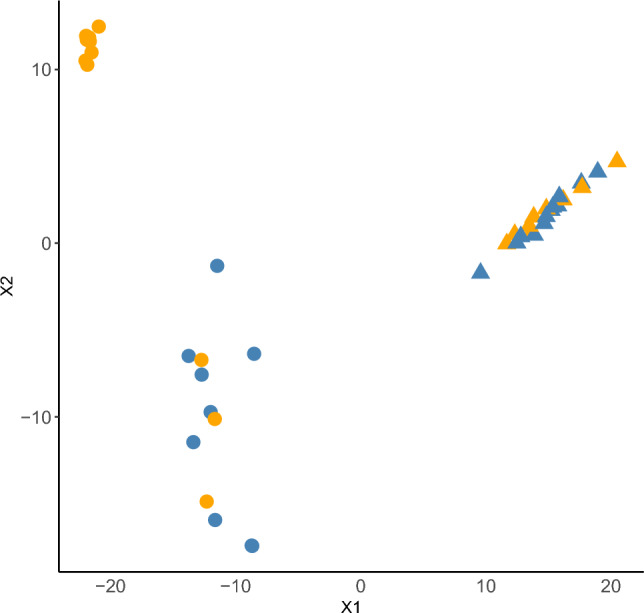
Visualisation of the first (X1) and second (X2) components of the semi-supervised KODAMA analysis. Controls are encoded using points, and triangles encode DM II individuals; high dietary fiber intake is colored blue, and low dietary fiber intake is colored orange.

To verify that differences in fiber intake have more impact in the control group and that the effect in the DM II group is rather low, we applied a combined unsupervised and semi-supervised learning approach (knowledge discovery by accuracy maximization, KODAMA on the same data as was used for the correlation and the multi-omics factor analysis. First, we ran KODAMA unsupervised to find the optimal number of parameters based on the lowest entropy. The optimal number of parameters that characterizes the data was 8 (*Entropy* = 6.8694). Then, KODAMA was applied semi-supervised on stratified data (stratified by disease) with the number of parameters set to 8. The results were very similar to the MOFA2 results. Using linear regression, we correlated the effect of diet on X1 and X2. In the control group, we found a significant influence of fiber intake on X1 and X2 (X1: *R*_*2*_^*adj*^ = 0.5492, *p* = 1.1 × 10^–4^; X2: *R*_*2*_^*adj*^ = 0.4508, *p* = 7.1 × 10^–4^), whereas this was not the case in the DM II group (X1: *R*_*2*_^*adj*^ = − 0.0518, *p* = 0.9011; X2: *R*_*2*_^*adj*^ = − 0.0455, *p* = 0.7219) (Fig. 4).

### Feature selection using machine learning

Based on the data described in the previous section, we tried to predict whether a sample is on HFD or on LFD. This prediction was made by applying a gradient-boosting machine approach (1000 iterations, see Material and Methods for details) to each disease group separately. In the healthy group, prediction accuracy was 73.2% (± 20.9), with 15 features showing an average influence above 2%. Only one of these features was not a bacterial genus (Metabolite U8), and the top three features were *Prevotella*, *Gemmiger* and *Lachnoclostridium B*; all 15 features are shown in Suppl. Figure 12. In the disease group, accuracy was 33.6% (± 19.9), with 13 features having an influence above 2% on average. Besides the fungal genus *Saccharomyces cerevisiae unclassified* and the metabolite lactate all remaining features were bacteria genera (Suppl. Figure 13).

## Discussion

The human gut microbiome is linked to various cardiometabolic disorders, primarily type 2 diabetes mellitus (DM II). Unfavorable variation in gut microbiome composition, known as dysbiosis, plays a significant role in DM II pathogenesis and response to anti-diabetic medications^19,30,31^. Several studies have also suggested an association between changes in the gut microbiome and the metabolic signature that may alter the intestinal barrier and signaling events that contribute to insulin homeostasis^32,33^.

Our study attempted to link the dysbiotic gut microbiome (here bacteriome and mycobiome) with the metabolic signature in DM II among the Middle Eastern population. We accounted for diet as a major factor in influencing the gut microbiome composition, both the bacterial and fungal communities associated with DM II. Although fungal communities comprise a small percentage of the total gut microbiome, previous reports have shown that these small numbers of fungi have surprisingly strong effects on dulling inflammatory responses in the gut^34,35^. Also, others have reported their impact on the bacterial community composition^36,37^. Interestingly, we did detect a significant influence on community composition (beta diversity) of dietary fiber intake (low or high) in the bacterial, fungal, and metabolite assay among DM II and healthy subjects. The observed dissimilarity of the alpha- and beta-diversity in correlation with the metabolic signature suggests an important difference in fungal composition and the corresponding metabolic profile at the intra-individual variability level and is consistent with previous publications^35,38^. In contrast, the inter-individual differences between the samples show that most of the samples are grouped as two different clusters that correspond to DM II and healthy individuals, highlighting an intriguing contrast in bacterial microbiota composition and metabolic profile, particularly, *Prevotella* abundances that showed a significant increase in the LFD group and consistent with the previous publication^28^. Fiber intake impacts *Prevotella* abundance which aids in polysaccharide breakdown. In our previous study, we also demonstrated enrichment in *Prevotella* with LFD, consistent with a significant increase in carbohydrate degradation modules observed in the GMM module analyses.

Despite the gut microbiome's resilience, studies repeatedly show that nutrition continues to have a significant impact on both its makeup and function^39,40^. The variety and abundance of microbial taxa are shaped by short- and long-term food patterns, which also have an impact on the metabolic activities of microbes^41^. Although the gut microbiome can adjust to dietary changes to preserve stability, long-term or drastic dietary changes can still upset its homeostasis. As a result, supporting a healthy gut flora and overall well-being requires a balanced, varied diet that contains a suitable number of fiber-rich foods.

Next, differential abundance testing determined the composition in relation to diet and determined the several bacterial genera such as the following; *Coprococcus A*, and *Sutterella* among others showed a significant increase in the HFD samples, whereas *Acidaminococcus, Bifidobacterium*, and *Prevotella* among others were higher abundant in the LFD samples when considering the control group. In the fungal community, no genus was significantly different between HFD and LFD in the healthy cohort. The metabolites Propionate, U8, and X2 Hydroxybutyrate were substantially lower in HFD, and X3 Hydroxyphenyl acetate was significantly higher in HFD compared to LFD. The data were consistent with findings in a previous western cohort^42,43^. Interestingly, however, the unidentified (U) metabolite U8 was found to be correlated with childhood overweight and rapid growth^44^. Moreover, a previous study determined that imidazole propionate is increased in diabetes and associated with dietary patterns and altered microbial ecology, particularly with low *Bacteroides* 2 enterotype, which has previously been associated with obesity^45^. Furthermore, hydroxyphenyl acetate, a phenylalanine breakdown product, has been associated with decreased insulin secretion and diabetes^46^. In order to identify the linkage between the gut microbiome and metabolome data, we applied several approaches in a two-dimensional relationship, such as Spearman rank correlations. We found that the two diets showed markedly different patterns of correlations, regardless of the disease group. Also, patterns between the control and DM II samples were different. Particularly, the correlations between bacterial and fungal data were dominated by negative correlations between *Meridosma* and several fungal genera in healthy LFD samples. In contrast, DM II LFD correlations were dominated by positive associations of *Odoribacter* with several fungal genera (e.g., *Humicola uncl.*, *Macrophomina phaseolina*, *Microascus manginii uncl.*, *Rhodotorula mucilaginosa*). The increasing abundance of *Odoribacter* has been previously reported to reduce plasma glucose and insulin levels and improve energy metabolism^47,48^. Furthermore, Metformin influences the abundance of several microbial taxa, particularly reducing the abundance of *Clostridium* spp. As well as the production of propionate and butyrate^49,50^. Intriguingly, we reported that the HFD DM II group showed marked negative correlations between *Odoribacter* and several fungal genera, further LFD DMII group showed marked negative correlations between Propionate and several bacterial genera. In the previous study, we determined that the phylum *Lentisphaera* and the genus *Phascolarctobacterium* have also been associated with the T2DM group and described a significant increase in individuals consuming a gluten-free diet. We also reported the genus *Odoribacter*, which includes butyrate-producing bacteria that negatively correlate with the T2DM group and also decrease in response to pre-natal metformin**.** Applying a multi-omics approach revealed that dietary fiber intake has a strong effect on the composition of the gut microbiome and metabolites, whereas other factors such as age, and BMI show no significant correlations. Interestingly, this effect is only present in the complete data and the healthy control samples, but not in the DM II group. Potentially, the impact of dietary fiber intake plays only a minor role compared to the dysbiosis in the gut microbiome due to Type II Diabetes Mellitus^51^. This finding was confirmed by a second unsupervised learning approach (KODAMA). Furthermore, a machine learning classifier, which was individually trained on each data set, performed better in the healthy group (accuracy 73.2%) compared to the disease group (33.6%) completing the picture that the effect of dietary fiber intake on the gut microbiome/metabolites is stronger in healthy individuals. However, even in the healthy group, the overall accuracy is not very high, most likely due to the small sample size. We acknowledge the small sample size as a potential limitation of this study and more advanced functional studies to validate some of these findings as potential biomarkers.

In conclusion, this study provides valuable insight into the links between dietary fiber intake and the gut microbiome, including fungal communities, and the metabolomic profiles in healthy and DM II subjects in the Middle East. In addition, we interrogated high vs. low fiber diet as a significant factor influencing the gut bacterial and fungal communities associated with healthy and DM II subjects. These aspects will be crucial in understanding the functional role of the gut microbiome and its alterations to support host homeostasis against metabolic and inflammatory disorders such as DM II. For example, *Prevotella* abundance showed a significant increase in the LFD group. Interestingly, the identified metabolites linked with the gut microbiome, such as U8, propionate, and hydroxyphenyl acetate, were previously connected with childhood overweight and increased diabetes, underscoring an important link between dietary patterns and altered gut microbiome communities. Perhaps these findings open the possibility for future follow-up studies to validate biomarkers responsible for or associated with the diseases towards precision medicine.

### Supplementary Information

Supplementary Information.

## Data Availability

ITS2 and 16S rRNA gene sequencing data used for this study were submitted to the European Nucleotide Archive (ENA) and are available under accession number PRJEB59916. Additional data supporting this study's findings are available on request from the corresponding author.
