# Supplementary material for: Uncovering the relationship between gut microbial dysbiosis, metabolomics, and dietary intake in type 2 diabetes mellitus and in healthy volunteers: a multi-omics analysis
Source: Sci Rep. 2023 Oct 20;13:17943. doi: 10.1038/s41598-023-45066-7 (PMC10589304; doi:10.1038/s41598-023-45066-7)
Supplement: Supplementary file 1 — Supplementary Information. [file 41598_2023_45066_MOESM1_ESM.pdf]

Supplementary Material

Sample	Status	Gender	Age	Fiber content	BMI
C003	Healthy	Female	34	High	35.2
C004	Healthy	Female	48	High	21.8
C005	Healthy	Male	22	High	23
C007	Healthy	Male	40	Low	25.7
C009	Healthy	Female	31	High	22.7
C010	Healthy	Female	21	High	18
C011	Healthy	Female	27	Low	25.4
C013	Healthy	Female	20	High	19.1
C014	Healthy	Female	32	High	26.1
C016	Healthy	Female	29	High	20.8
C017	Healthy	Female	29	Low	18.4
C018	Healthy	Female	20	Low	23.3
C019	Healthy	Female	20	High	22.2
C020	Healthy	Female	30	Low	26.1
C021	Healthy	Female	29	Low	24.7
C022	Healthy	Female	26	Low	24.6
C023	Healthy	Female	22	Low	24.6
C024	Healthy	Female	21	Low	24.8
C025	Healthy	Female	20	Low	22.9
C026	Healthy	Female	21	Low	39.3
P005	T2D	Female	62	High	23.6
P006	T2D	Male	48	Low	28.4
P008	T2D	Female	67	High	34.9
P009	T2D	Female	64	High	41.8
P010	T2D	Female	67	Low	20.3
P011	T2D	Male	38	High	47.2
P015	T2D	Female	69	Low	28.3
P017	T2D	Female	70	Low	27.2
P018	T2D	Female	49	High	24.9
P019	T2D	Female	62	Low	38.8
P020	T2D	Female	66	Low	34.3
P030	T2D	Male	64	Low	27.2
P033	T2D	Female	46	High	31.2
P035	T2D	Male	57	High	30.4
P036	T2D	Male	42	High	32.3
P037	T2D	Female	54	High	27.3
P038	T2D	Female	65	Low	36.3
P040	T2D	Female	58	Low	31.2
P043	T2D	Female	79	High	19.8
P045	T2D	Female	65	High	27
P046	T2D	Male	76	High	34.7

Supplementary Table 1: Demographic information such as age, gender, BMI and diet.

Variable	Df	SumOfSqs	R2	F	p value
Diet	1	3869.1190	0.0487	2.20914	0.00222
Disease	1	4164.0980	0.0524	2.377563	0.00083
Prevotella	1	5196.2720	0.0654	2.966901	0.00003
Age	1	1717.8480	0.0216	0.980835	0.45324
Gender	1	2140.9880	0.0269	1.222434	0.17083
BMI	1	2019.3730	0.0254	1.152996	0.22804
Diet:Disease	1	2573.1670	0.0324	1.469194	0.05975
Residual	33	57796.6700	0.7272	NA	NA
Total	40	79477.5360	1.0000	NA	NA

Supplementary Table 2: Statistical assessment of beta diversity for bacterial assay. Shown are the results from the PERMANOVA for each variable (Df: degrees of freedom; SumOfSqs: Sum of squares); p-values were calculated using 99,999 permutations.

Variable	Df	SumOfSqs	R2	F	p value
Diet	1	909.0071	0.0443	1.9392204	0.00186
Disease	1	1100.8902	0.0536	2.3485721	0.00012
Prevotella	1	762.2941	0.0371	1.6262319	0.01293
Age	1	620.2533	0.0302	1.3232107	0.07337
Gender	1	621.5849	0.0303	1.3260515	0.07772
BMI	1	420.0900	0.0205	0.8961945	0.66302
Diet:Disease	1	619.0827	0.0302	1.3207134	0.06434
Residual	33	15468.7078	0.7538	NA	NA
Total	40	20521.9102	1.0000	NA	NA

Supplementary Table 3: Statistical assessment of beta diversity for fungal assay. Shown are the results from the PERMANOVA for each variable (Df: degrees of freedom; SumOfSqs: Sum of squares); p-values were calculated using 99,999 permutations.

Variable	Df	SumOfSqs	R2	F	p value
Diet	1	318.08996	0.0568	2.7250	0.00356
Disease	1	731.77344	0.1307	6.2690	0.00001
Prevotella	1	106.30849	0.0190	0.9107	0.52307
Age	1	99.95702	0.0179	0.8563	0.58915
Gender	1	173.50165	0.0310	1.4864	0.10733
BMI	1	121.8079	0.0218	1.0435	0.37561
Diet:Disease	1	195.218	0.0349	1.6724	0.06275
Residual	33	3852.04909	0.6880	NA	NA
Total	40	5598.70555	1.0000	NA	NA

Supplementary Table 4: Statistical assessment of beta diversity for metaboliote assay. Shown are the results from the PERMANOVA for each variable (Df: degrees of freedom; SumOfSqs: Sum of squares); p-values were calculated using 99,999 permutations.

Assay	Comparision	Genus/Metabolite	ALDEx2	ANCOM-BC	MaAsLin2	dacomp	Score
Bacteriome	Control	Bifidobacterium	TRUE	TRUE	FALSE	TRUE	3
		Phocaeicola	TRUE	TRUE	TRUE	TRUE	4
		Prevotella	TRUE	TRUE	TRUE	TRUE	4
		Barnesiella	FALSE	TRUE	FALSE	FALSE	1
		GCA-900199385	FALSE	TRUE	FALSE	FALSE	1
		Bariatricus	FALSE	TRUE	FALSE	FALSE	1
		Blautia_A	FALSE	FALSE	FALSE	TRUE	1
		CAG-317	TRUE	TRUE	FALSE	TRUE	3
		Choladocola	FALSE	TRUE	FALSE	FALSE	1
		Coprococcus_A	FALSE	TRUE	TRUE	TRUE	3
		Dorea	FALSE	TRUE	FALSE	FALSE	1
		Dorea_A	TRUE	TRUE	FALSE	FALSE	2
		Lachnoclostridium_B	FALSE	TRUE	TRUE	FALSE	2
		Lachnospira	FALSE	TRUE	TRUE	TRUE	3
		Eubacterium_R	FALSE	TRUE	FALSE	FALSE	1
		Ruminococcus_E	FALSE	TRUE	FALSE	FALSE	1
		Dysosmobacter	FALSE	TRUE	FALSE	TRUE	2
		Gemmiger	TRUE	TRUE	TRUE	TRUE	4
		Ligilactobacillus	TRUE	TRUE	TRUE	TRUE	4
		Acidaminococcus	TRUE	TRUE	TRUE	TRUE	4
		Mitsuokella	FALSE	TRUE	TRUE	TRUE	3
		Mesosutterella	TRUE	TRUE	TRUE	TRUE	4
		Sutterella	TRUE	TRUE	TRUE	TRUE	4
		CAG.317	FALSE	FALSE	TRUE	FALSE	1
Mycobiome	Control	Candida_inconspicua_unclassified	FALSE	TRUE	FALSE	FALSE	1
		Candida_albicans_unclassified	FALSE	TRUE	FALSE	FALSE	1
		Basidiomycota_unclassified	FALSE	TRUE	FALSE	FALSE	1
		Malassezia_restricta	FALSE	TRUE	FALSE	FALSE	1
Metabolites	Control	X3.Hydroxyphenylacetate	FALSE	TRUE	TRUE	NA	2
		Arabinose	FALSE	TRUE	FALSE	NA	1
		Galactose	FALSE	TRUE	FALSE	NA	1
		U10.Unknown.sugar	FALSE	TRUE	FALSE	NA	1
		U4	FALSE	TRUE	FALSE	NA	1
		U7	FALSE	TRUE	FALSE	NA	1
		U8	FALSE	TRUE	TRUE	NA	2
		Aspartate	FALSE	TRUE	FALSE	NA	1
		Proprionate	FALSE	TRUE	TRUE	NA	2
		X2.Hydroxybutyrate	FALSE	TRUE	TRUE	NA	2

Supplementary Table 5: Differentially different abundant taxa/metabolites per assay and comparison. TRUE/FALSE refers to whether a method detected a particular genus/metabolite as differentially

abundant (fdr < 0.1); dacomp was not run for metabolite assay. The ‘Score’ column shows the total weight for each genus/metabolite.

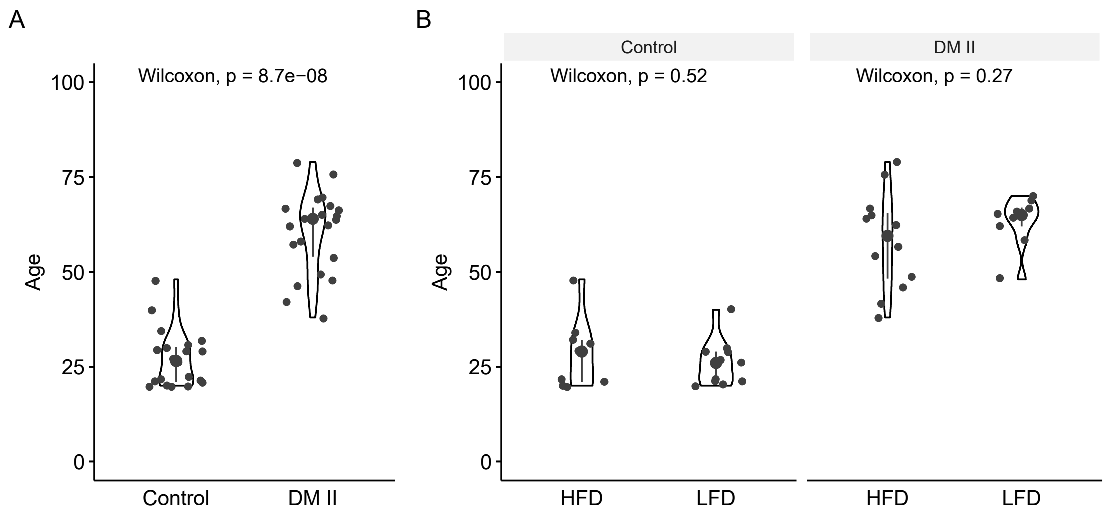

Supplementary Figure 1: Violin plots showing age distribution across control and DM II samples (A) and across dietary fiber intake (HFD = high fiber intake; LDF = low fiber intake) stratified by disease (B).

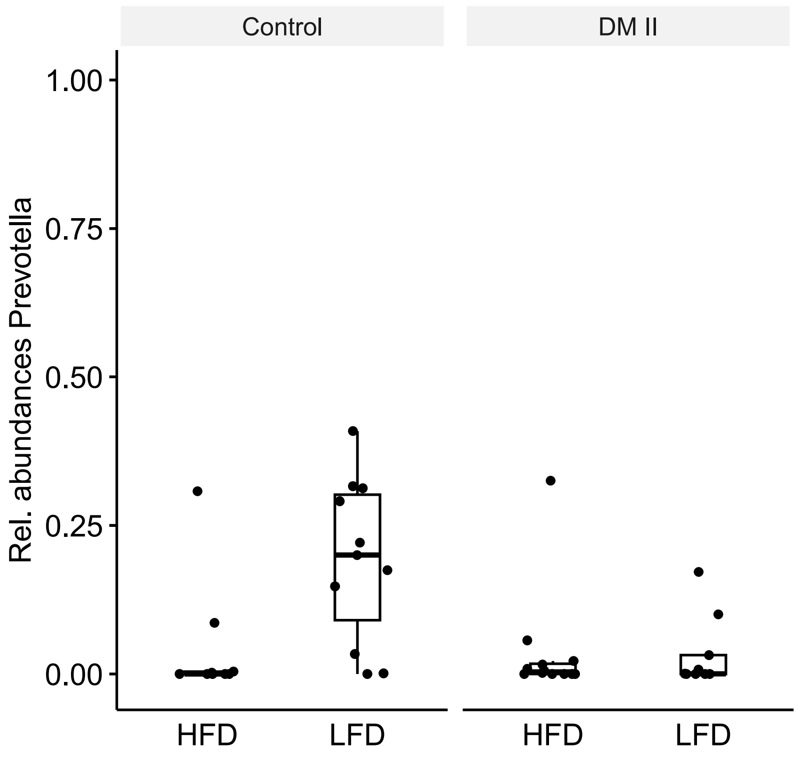

Suppl. Figure 2: Relative abundances of the genus *Prevotella* for high and low dietary fiber intake stratified by healthy controls and DM II samples.

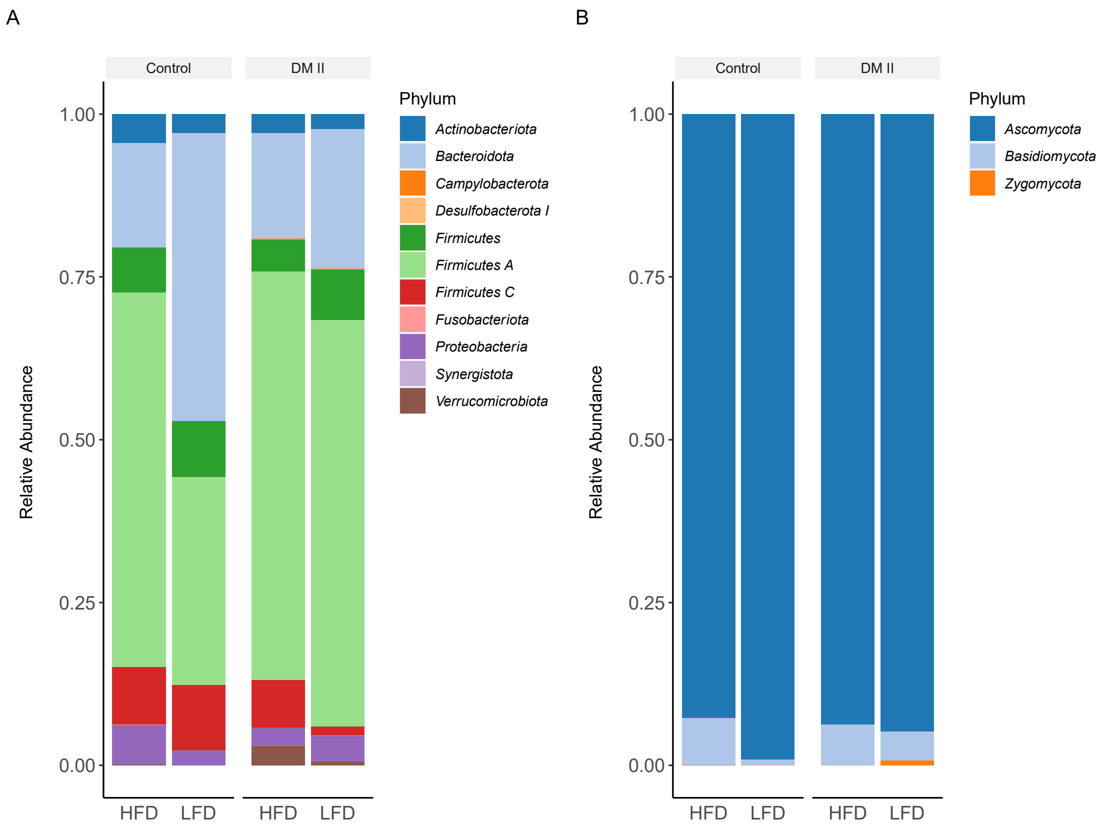

Suppl. Figure 3: Phyla abundances of the bacteriome (A) and mycobiome (B) for healthy controls and DM II samples.

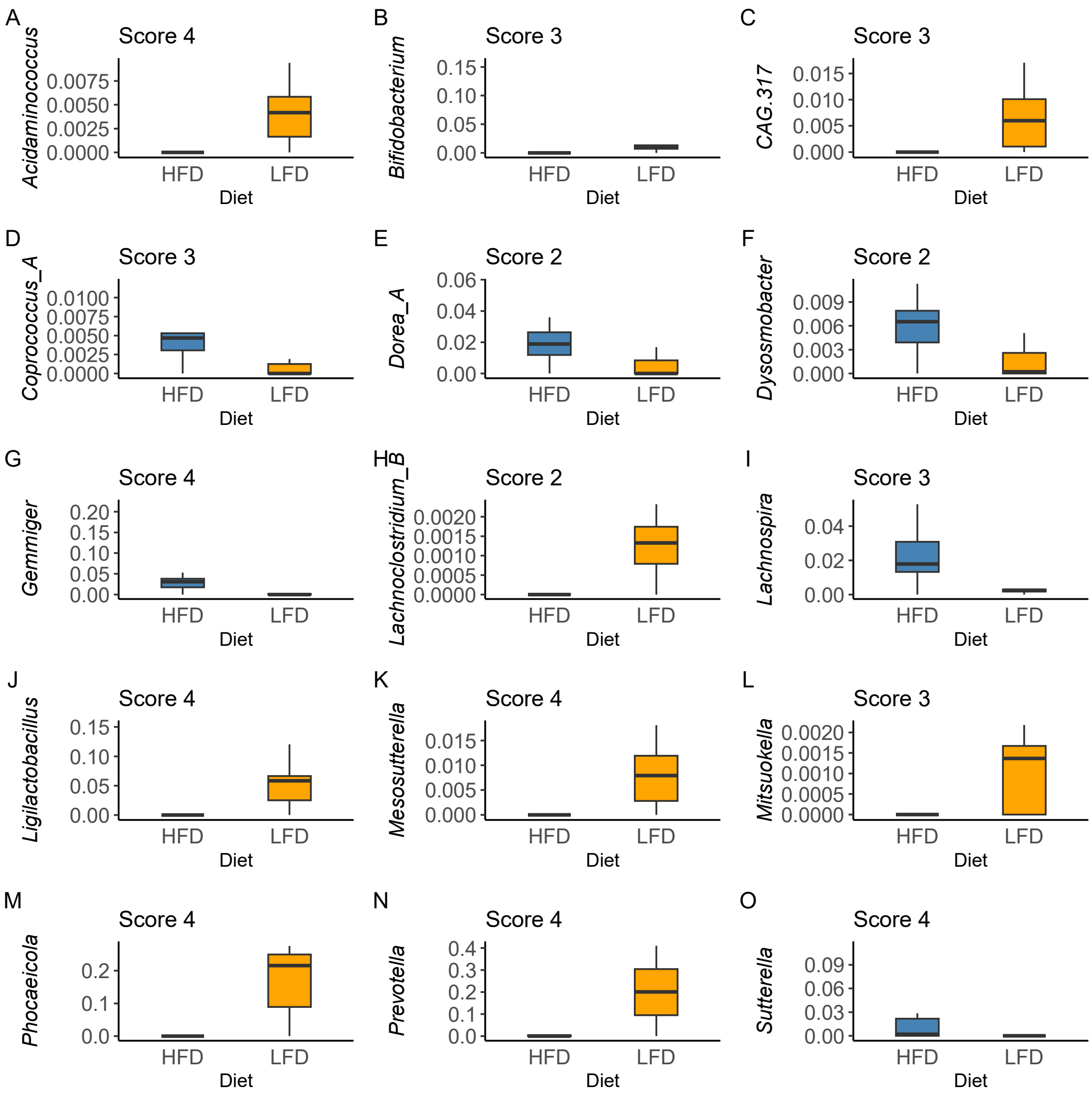

Suppl. Figure 4: Relative abundances of significantly different bacterial genera (panels A-O) with respect to dietary fiber intake in healthy controls. Score refers to the number of approaches identifying a particular genus as significantly different (*fdr* < 0.1).

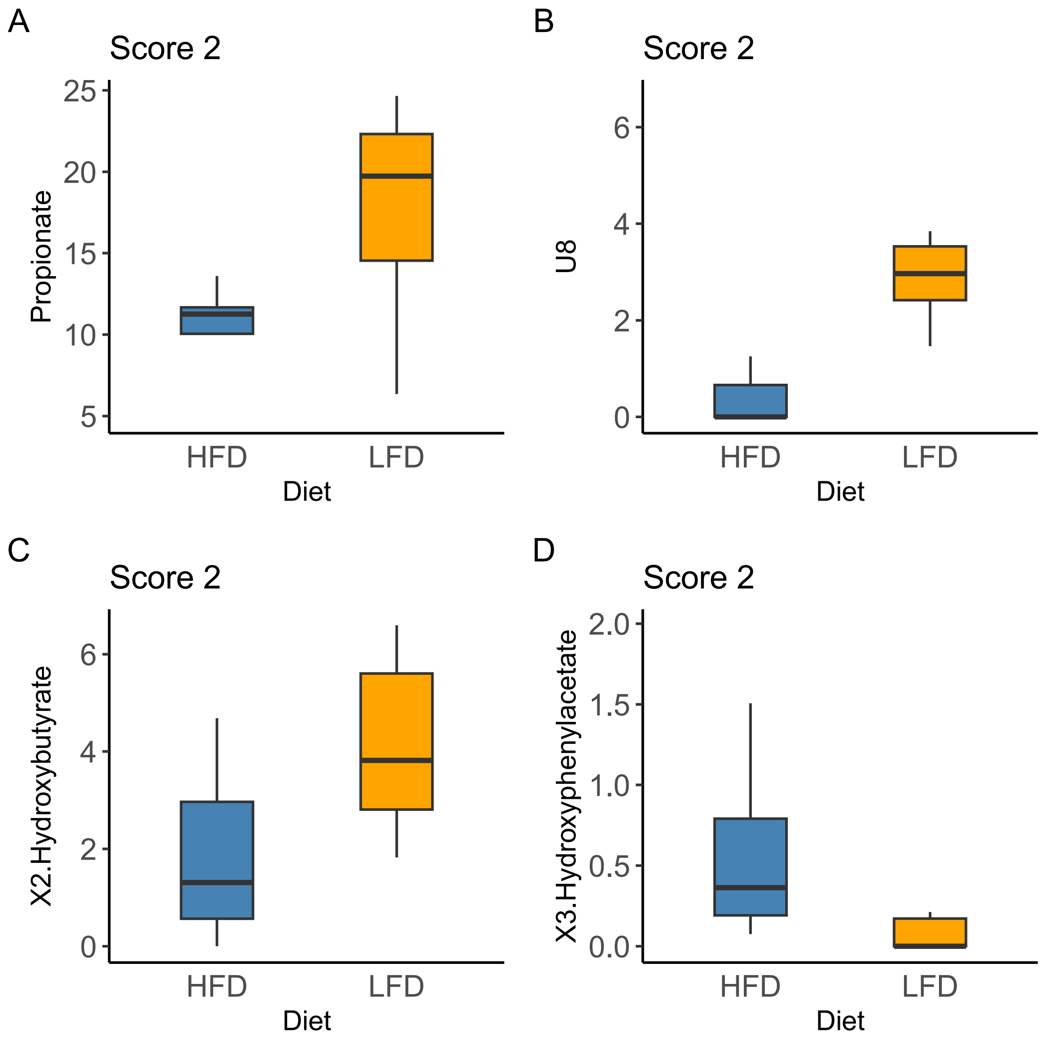

Suppl. Figure 5: Significantly different metabolites (panels A-D) with respect to dietary fiber intake in healthy controls. Score refers to the number of approaches identifying a particular metabolite as significantly different (*fdr* < 0.1).

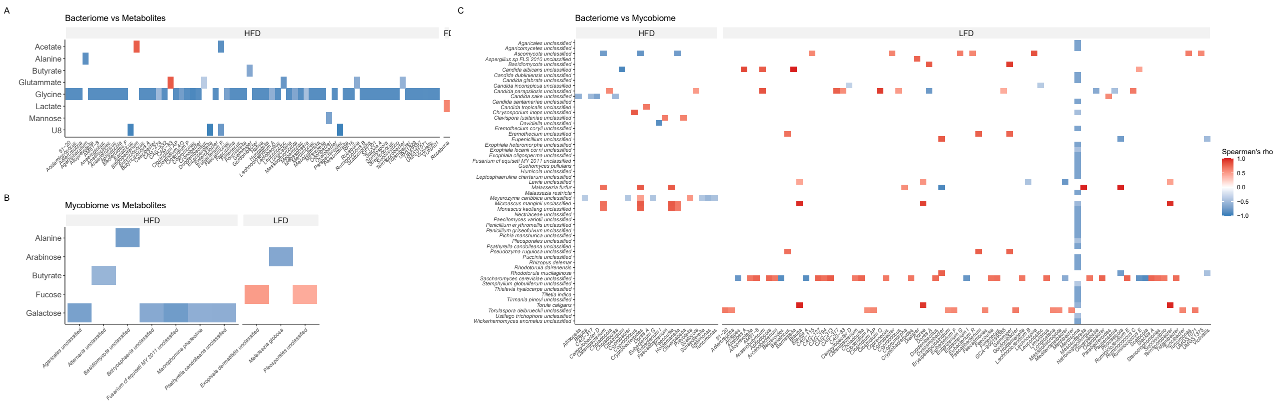

Suppl. Figure 6: Spearman's rank correlations in controls stratified by dietary fiber intake between bacteriome and metabolites (A), mycobiome and metabolites (B), and bacteriome and mycobiome (C). Only significant correlations ($p < 0.01$, absolute $\rho > 0.3$) are shown; positive correlations are shown in red, and negative correlations in blue.

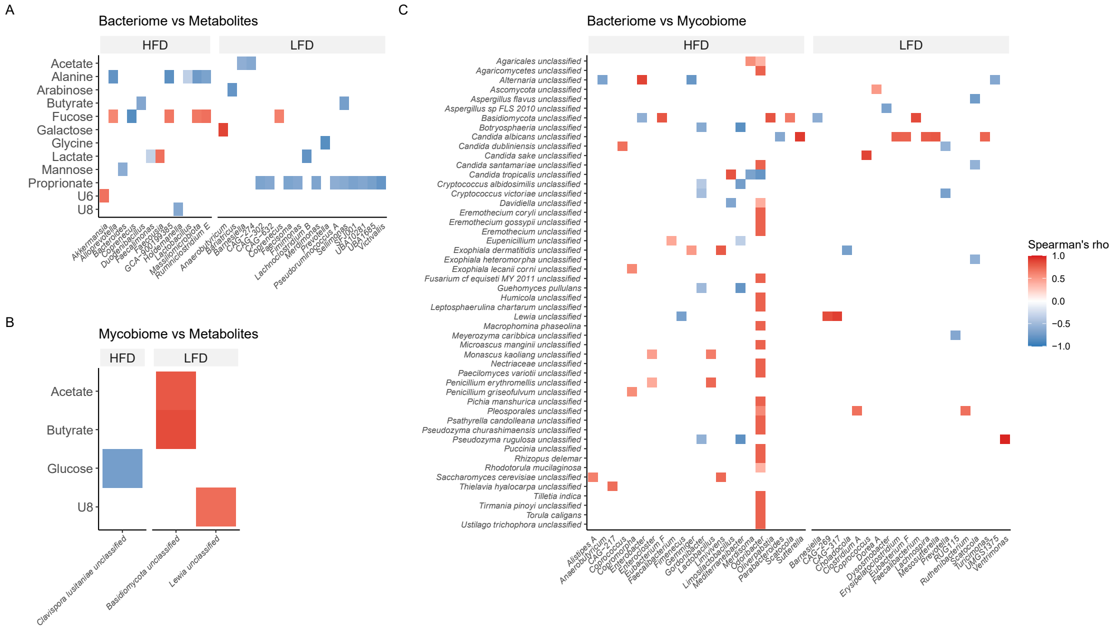

Suppl. Figure 7: Spearman's rank correlations in DM II individuals stratified by fiber intake between bacteriome and metabolites (A), mycobiome and metabolites (B), and bacteriome and mycobiome (C). Only significant correlations ($p < 0.01$, absolute $\rho > 0.3$) are shown; positive correlations are shown in red, and negative correlations in blue.

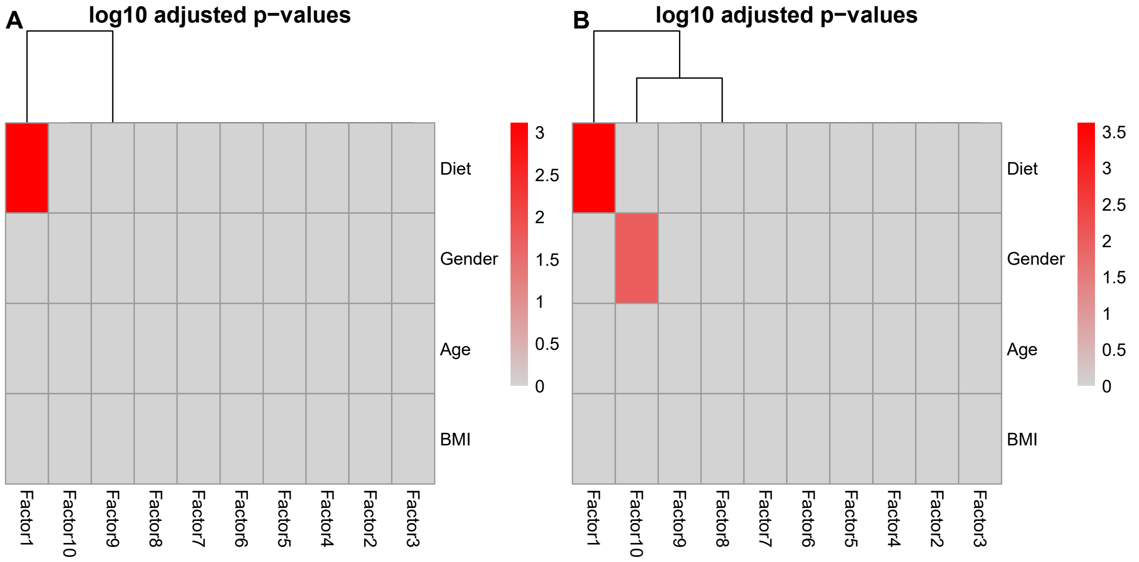

Suppl. Figure 8: Correlation of identified factors with selected covariates (only correlations where $p_{adj} < 0.01$ are shown; adjusted *p*-values were $\log_{10}$ transformed) for the full data (A) and the healthy control samples (B).

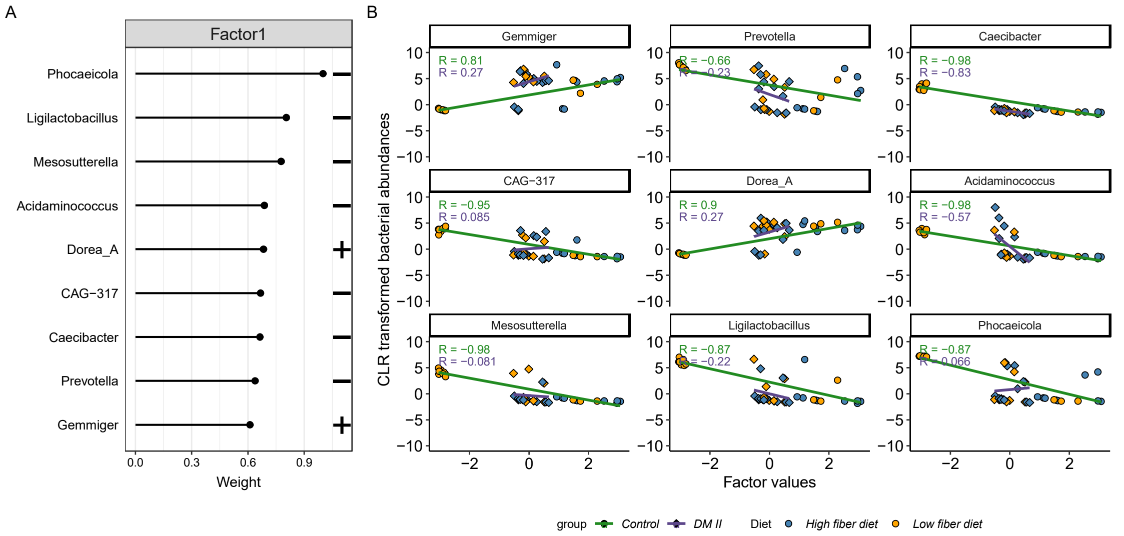

Suppl. Figure 9: Weights for bacterial genera retrieved from the multi-omics factor analysis for Factor1 with an absolute weight above 0.6 (A). (B) Scatterplot with *clr* transformed abundance values where points denote healthy controls and squares denote DM II samples; regression lines and results from linear regression are shown for healthy controls (green) and DM II samples (dark magenta). High dietary fiber intake is shown in blue, and low dietary fiber intake is shown in orange.

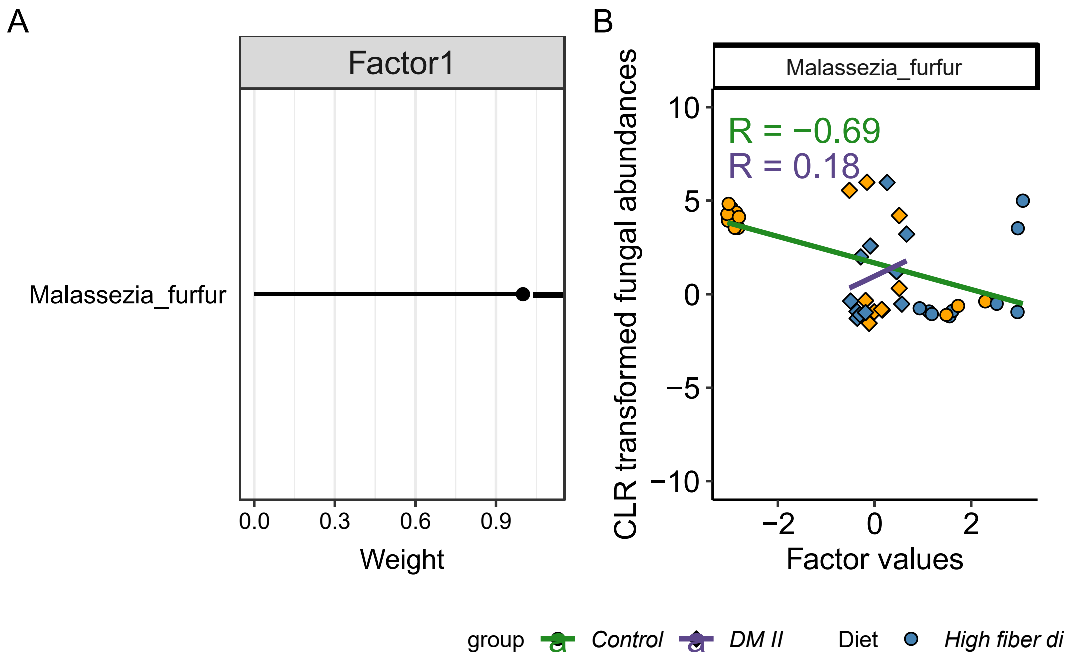

Suppl. Figure 10: Weights for fungal genera retrieved from the multi-omics factor analysis for Factor1 with an absolute weight above 0.6 (A). (B) Scatterplot with *clr* transformed abundance values where points denote healthy controls and squares denote DM II samples; regression lines and results from linear regression are shown for healthy controls (green) and DM II samples (dark magenta). High dietary fiber intake is shown in blue, and low dietary fiber intake is shown in orange.

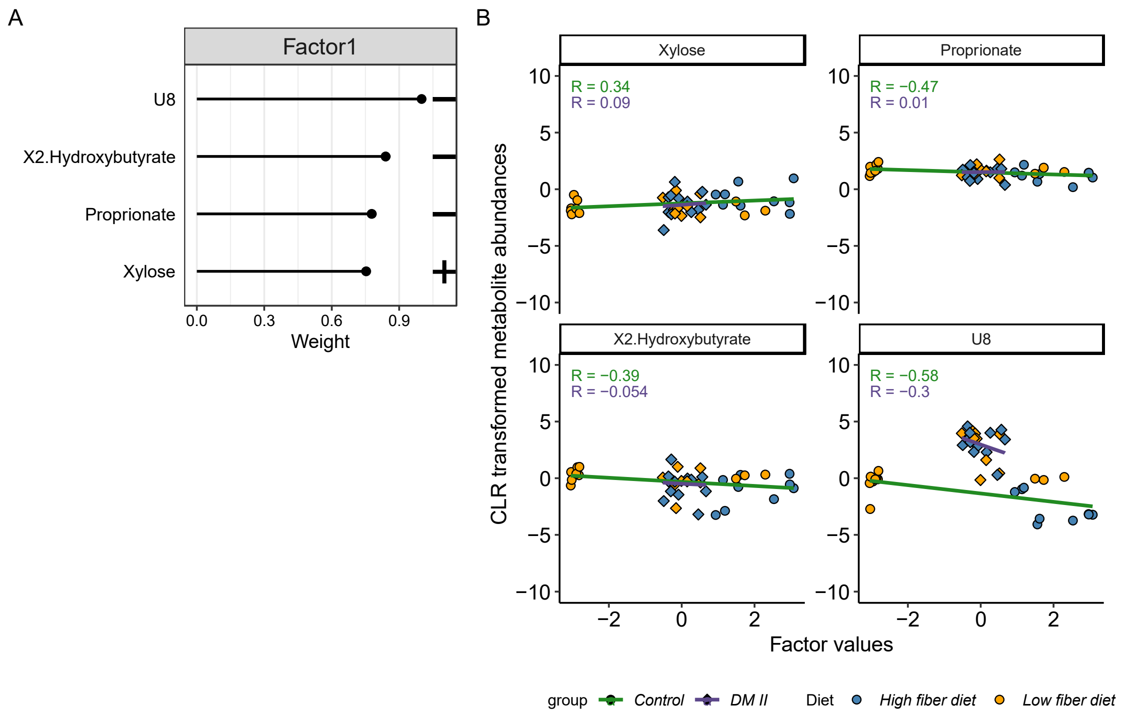

Suppl. Figure 11: Weights for metabolites retrieved from the multi-omics factor analysis for Factor1 with an absolute weight above 0.6 (A). (B) Scatterplot with *clr* transformed abundance values where points denote healthy controls and squares denote DM II samples; regression lines and results from linear regression are shown for healthy controls (green) and DM II samples (dark magenta). High dietary fiber intake is shown in blue, and low dietary fiber intake is shown in orange.

Healthy: Gradient Boosting Machine (1,000 iterations)

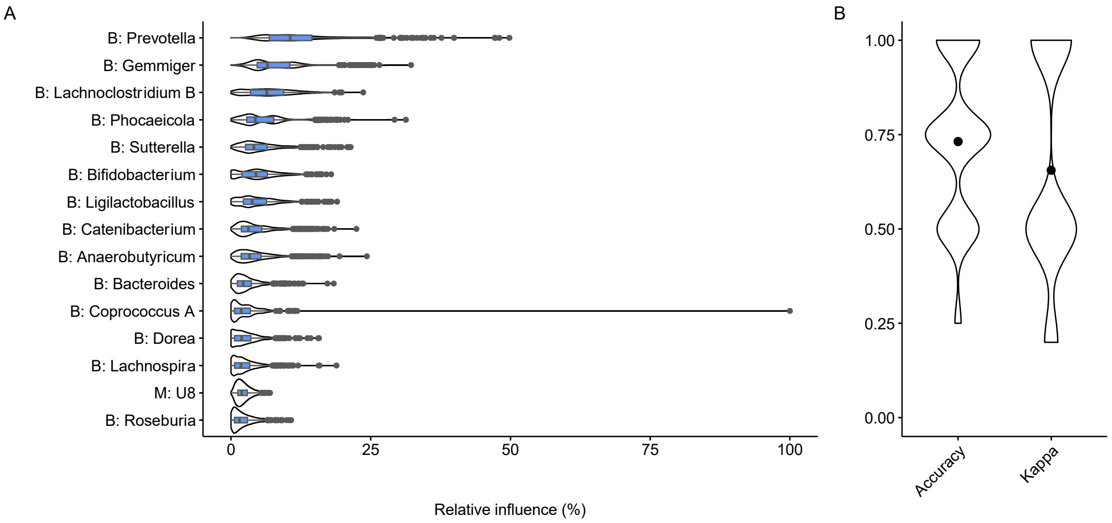

Suppl. Figure 12: The classification of healthy volunteers into HFD and LFD was run for 1,000 iterations selecting a train and a test set each time. Top features of the gradient boosting machine algorithm (influence above 2%) (A). Panel (B) shows the distribution and the average Accuracy and Kappa for the 1,000 iterations of the prediction.

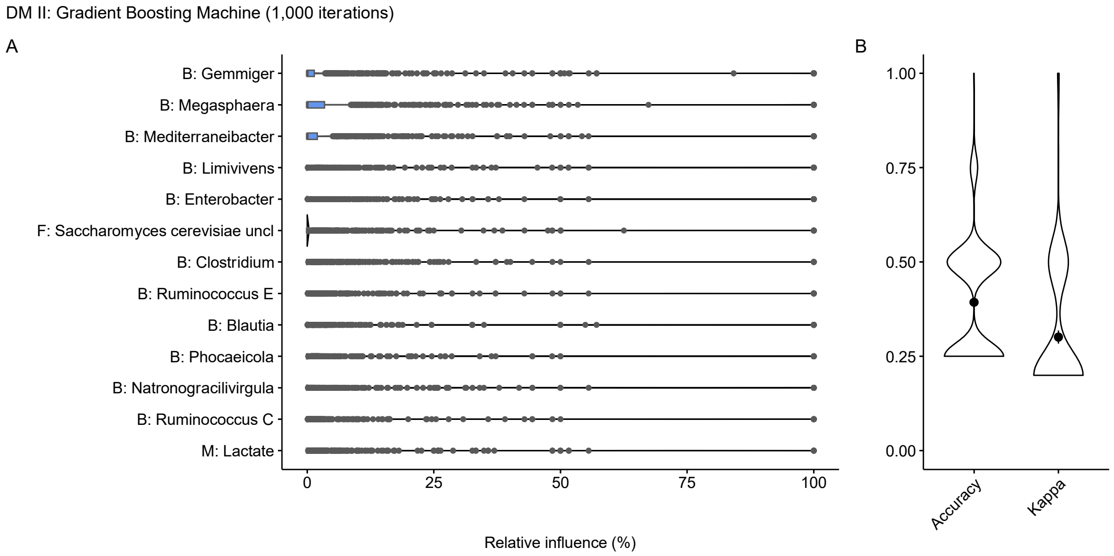

Suppl. Figure 13: The classification of the DM II samples into HFD and LFD was run for 1,000 iterations selecting a train and a test set each time. Top features of the gradient boosting machine algorithm (influence above 2%) (A). Panel (B) shows the distribution and the average Accuracy and Kappa for the 1,000 iterations of the prediction.
